# Supplementary material for: Sharing Vitamin B12 between Bacteria and Microalgae Does Not Systematically Occur: Case Study of the Haptophyte Tisochrysis lutea
Source: Microorganisms. 2022 Jul 1;10(7):1337. doi: 10.3390/microorganisms10071337 (PMC9323062; doi:10.3390/microorganisms10071337)
Supplement: Supplementary file 1 [file microorganisms-10-01337-s001.zip › microorganisms-1779056-supplementary/Supplementary_Figures.pdf]

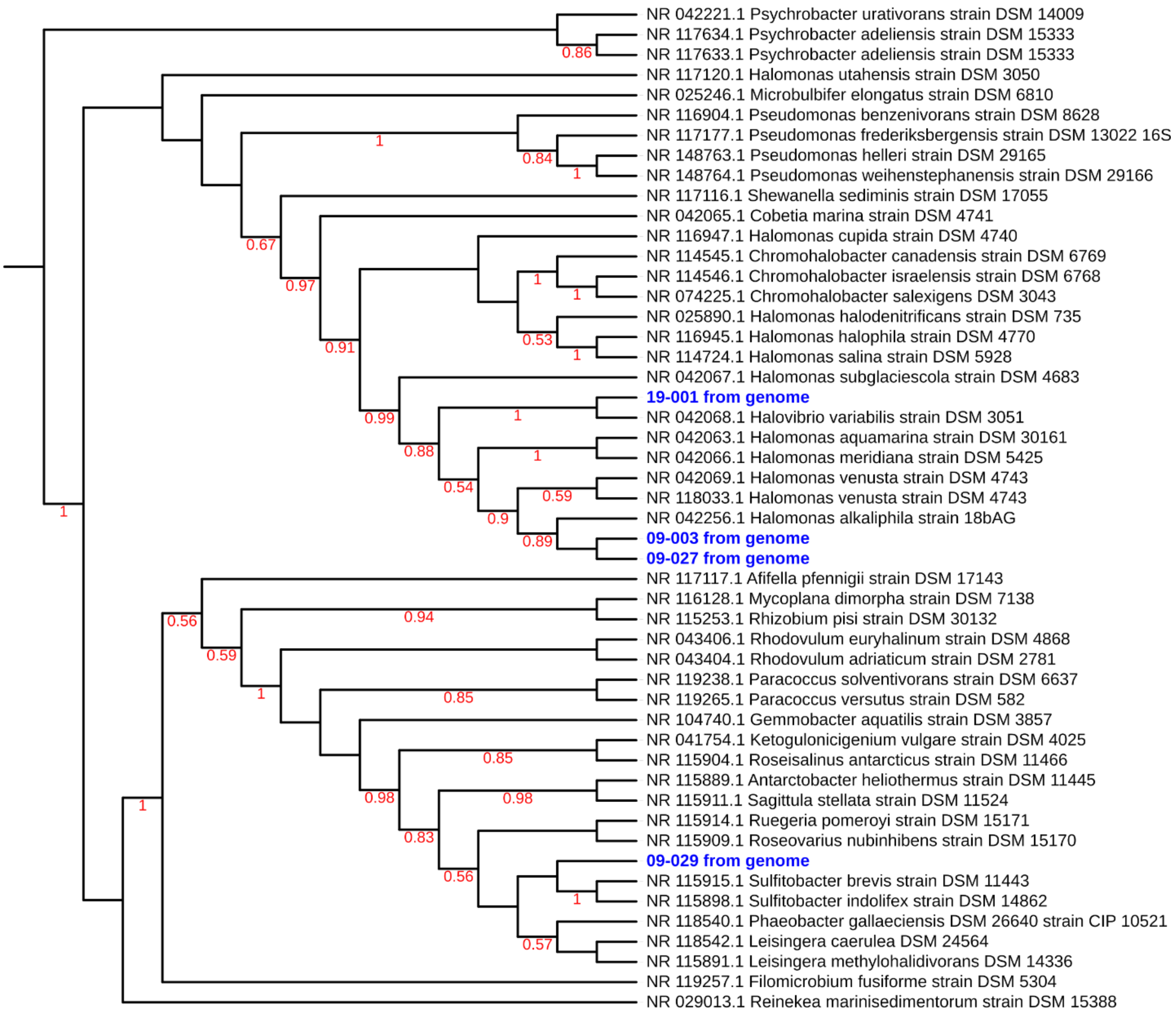

Supplementary Figure S1. 16S rDNA phylogenetic tree for reference (black) and selected (blue) bacteria inferred from Maximum Likelihood (100 bootstrap). Bootstrap values superior to 0.5 are indicated.

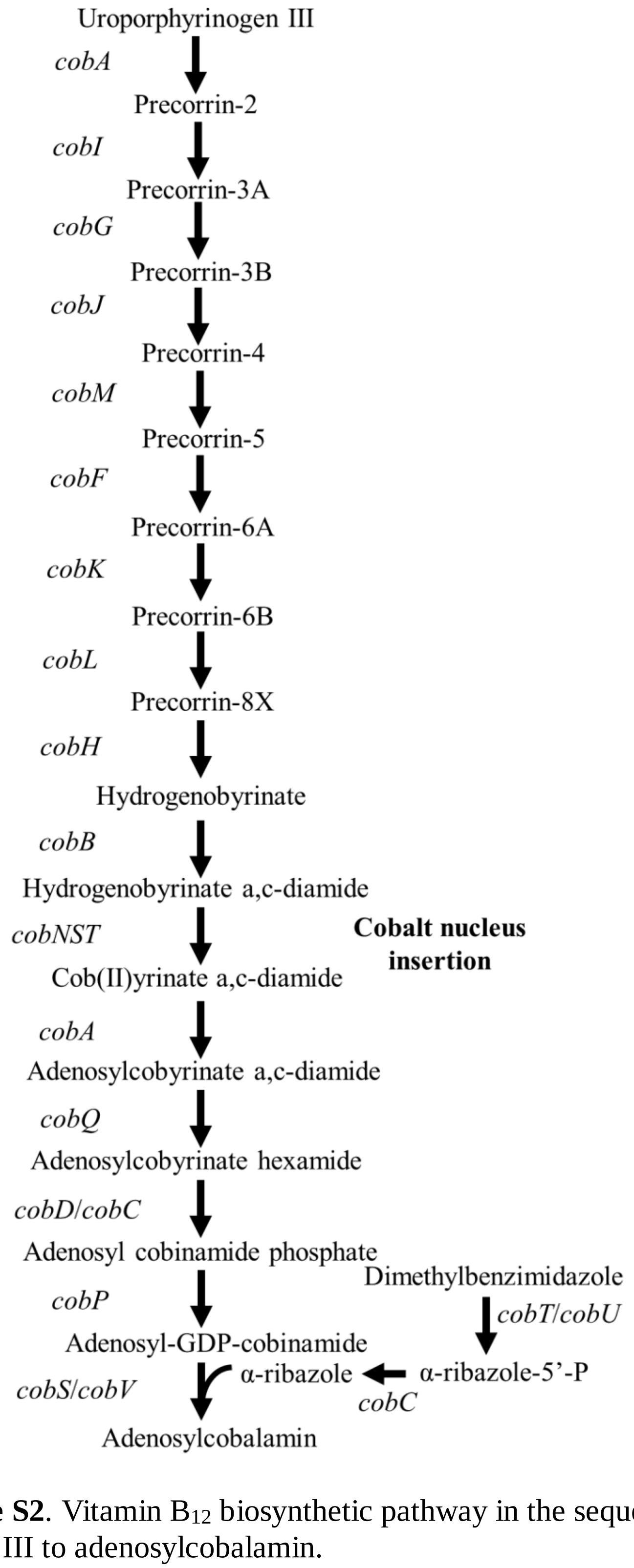

Supplementary Figure S2. Vitamin B_{12} biosynthetic pathway in the sequenced bacteria, starting from uroporphyrinogen III to adenosylcobalamin.
